# A parallel multisplitting method with self-adaptive weightings for solving *H*-matrix linear systems

**DOI:** 10.1186/s13660-017-1370-7

**Published:** 2017-05-01

**Authors:** Ruiping Wen, Hui Duan

**Affiliations:** 1 Higher Education Key Laboratory of Engineering and Scientific Computing, Taiyuan Normal University, Taiyuan, Shanxi 030012 P.R. China; 2 Department of Mathematics, Taiyuan Normal University, Taiyuan, Shanxi 030012 P.R. China

**Keywords:** linear systems, self-adaptive weightings, convergence, parallel multisplitting, *H*-matrix

## Abstract

In this paper, a parallel multisplitting iterative method with the self-adaptive weighting matrices is presented for the linear system of equations when the coefficient matrix is an *H*-matrix. The zero pattern in weighting matrices is determined in advance, while the non-zero entries of weighting matrices are determined by finding the optimal solution in a hyperplane of *α* points generated by the parallel multisplitting iterations. Especially, the nonnegative restriction of weighting matrices is released. The convergence theory is established for the parallel multisplitting method with self-adaptive weightings. Finally, a numerical example shows that the parallel multisplitting iterative method with the self-adaptive weighting matrices is effective.

## Introduction and preliminaries

To solve the large sparse linear system of equations

1.1 $$ Ax=b, \quad A\in\mathbf{R}^{n\times n} \mbox{ nonsingular and } b\in \mathbf{R}^{n}, $$

O’Leary and White [1] first proposed parallel methods based on multisplittings of matrices in 1985, where several basic convergence results may be found. A multisplitting of *A* is a collection of triples of $n\times n$ matrices $(M_{i},N_{i},E_{i})_{i=1}^{\alpha}$ ($\alpha\leq n$, a positive integer) with $M_{i},N_{i},E_{i}\in\mathbf{R}^{n\times n}$, and then the following method for solving system (1.1) was given:

$$\begin{aligned}& A=M_{i}-N_{i} ,\quad i=1,2,\ldots,\alpha, \\& M_{i}x_{i}^{(k)}=N_{i}x^{(k-1)}+b, \quad i=1,2,\ldots,\alpha; k=1,2,\ldots, \\& x^{(k)}=\sum_{i=1}^{\alpha}E_{i}x_{i}^{(k)}, \end{aligned}$$

where $M_{i}$, $i=1,2,\ldots,\alpha$, are nonsingular and $E_{i}=\operatorname{diag}(e_{1}^{(i)}, e_{2}^{(i)}, \ldots, e_{n}^{(i)})$, $i=1,2,\ldots,\alpha$, satisfy $\sum_{i=1}^{\alpha}E_{i}=I$ ($I\in\mathbf{R}^{n\times n}$ is the identity matrix). By the way, $x^{(0)}$ is an initial vector of $x_{*}=A^{-1}b$.

There has been a lot of study (see [1–13]) on the parallel iteration methods for solving the large sparse system of linear equations (1.1). In particular, when the coefficient matrix *A* is an *M*-matrix or an *H*-matrix, many parallel multisplitting iterative methods (see, e.g., [1–7, 10]) were presented, and the weighting matrices $E_{i}$, $i=1,2,\ldots,\alpha$, were generalized (see, e.g., [3, 4, 6–10])

1.2 $$ \sum_{i=1}^{\alpha}E_{i}^{(k)}=I \ (\mbox{or }\neq I),\quad E_{i}^{(k)}\geq0, \mbox{diagonal}, i=1,2,\ldots,\alpha; k=1,2,\ldots, $$

but these weighting matrices were preset as multi-parameter.

As we know, the weighting matrices play an important role in parallel multisplitting iterative methods, but the weighting matrices in all the above-mentioned methods are determined in advance, they are not known to be good or bad, and this influences the efficiency of parallel methods. Recently, Wen and co-authors [13] discussed self-adaptive weighting matrices for a symmetric positive definite linear system of equations; Wang and co-authors [11] discussed self-adaptive weighting matrices for non-Hermitian positive definite linear system of equations. In this paper, we focus on the *H*-matrix, which originates from Ostrowski [14]. The *H*-matrix is a class of the important matrices that has many applications. For example, numerical methods for solving PDEs are a source of many linear systems of equations whose coefficients form *H*-matrices (see [15–18]). Are self-adaptive weighting matrices true for the linear system of equations when the coefficient matrix is an *H*-matrix? We will discuss this problem in this paper.

Here, we generalize the weighting matrices $E_{i}$ ($i=1,2,\ldots,\alpha$) to $E_{i}^{(k)}$ ($i=1,2,\ldots,\alpha$; $k=1,2, \ldots$), which can be divided into two steps: each splitting is first dealt with by a different processor, the weighting matrices $E_{i}^{(k)}$ ($i=1,2,\ldots,\alpha$; $k=1,2,\ldots$) will mask large portion of the matrix, so that each processor deals with a smaller matrix; later, the weighting matrices $E_{i}^{(k)}$ ($i=1,2,\ldots,\alpha$; $k=1,2, \ldots$) will be approximated to ‘the best’ choices well for *k*-step iteration in the hyperplane generated by $\{x_{i}^{(k)}, i=1,2, \ldots, \alpha\}$, $k=1,2, \ldots $ . In this paper, we determine the weighting matrices $E_{i}^{(k)}$ ($i=1,2,\ldots,\alpha$; $k=1,2, \ldots$) for above reasons. Firstly, decreasing execution times is completed by the zero-entry’s set in weighting matrices. Secondly, the self-adaptive weighting matrices are reached by finding the ‘good’ point in the hyperplane generated by $\{ x_{i}^{(k)}, i=1,2, \ldots, \alpha\}$, $k=1,2, \ldots $ . Thus, the scheme of finding the self-adaptive weighting matrices is established for the parallel multisplitting iterative method, it has two advantages as follows:

- The weighting matrices are not necessarily nonnegative;
- Only one splitting of *α* splittings is required to be convergent.

In the rest of this study, we first give some notations and preliminaries in Section 1, and then a parallel multisplitting iterative method with the self-adaptive weighting matrices is put forward in Section 2. The convergence of the parallel multisplitting iterative method is established in Section 3. Moreover, we give the computational results of the parallel multisplitting iterative method by a problem in Section 4. We end the paper with a conclusion in Section 5.

Here are some essential notations and preliminaries. $\mathbf{R}^{n\times n}$ is used to denote the $n\times n$ real matrix set, and $\mathbf{R}^{n}$ is the *n*-dimensional real vector set. $A^{T}$ represents the transpose of the matrix *A*, and $x^{T}$ denotes the transpose of the vector *x*. $\langle A\rangle$ stands for the comparison (Ostrowski) matrix of the matrix *A*, and $|A|$ is the absolute matrix of the matrix *A*.

In what follows, when *A* is a strictly diagonally dominant matrix in column, *A* is called a strictly diagonally dominant matrix.

### Definition 1.1

[17]

The matrix *A* is an *H*-matrix if there exists a positive diagonal matrix *D* such that the matrix *DA* is a strictly diagonally dominant matrix.

### Property 1.2

[14]

The matrix *A* is an *H*-matrix if and only if $\langle A\rangle$ is an *M*-matrix.

### Definition 1.3

[8, 12]

Suppose that *A* is an *H*-matrix. Let $A=M-N$, which is called an *H*-compatible splitting if $\langle A\rangle =\langle M\rangle-|N|$.

### Property 1.4

[15]

Let *A* be an *H*-matrix, then $|A^{-1}|\le\langle A\rangle^{-1}$.

## Description of the method

Here, we present a parallel multisplitting iterative method with the self-adaptive weighting matrices.

Let

2.1 $$\begin{aligned}& A=M_{i}-N_{i}, \quad i=1,2,\ldots,\alpha. \end{aligned}$$

2.2 $$\begin{aligned}& E_{i}^{(k)}=\operatorname{diag}\bigl(e_{1}^{(i,k)}, \ldots,e_{n}^{(i,k)}\bigr),\qquad \sum _{i=1}^{\alpha}E_{i}^{(k)}=I, \quad k=1,2,\ldots. \end{aligned}$$

By introducing

2.3 $$ T_{i}=M_{i}^{-1}N_{i}, \quad i=1,2, \ldots,\alpha. $$

The iteration matrix of *k*th step

2.4 $$ T_{k}=\sum_{i=1}^{\alpha}E_{i}^{(k)}M_{i}^{-1}N_{i}= \sum_{i=1}^{\alpha }E_{i}^{(k)}T_{i}. $$

The preset nonzero set

2.5 $$ S(i,k)=\bigl\{ j|e_{j}^{(i,k)}\neq0\bigr\} , \qquad S(k)=\bigcup _{i=1}^{\alpha}S(i,k). $$

### Method 2.1

Step 0.: Given a tolerance $\epsilon>0$ and an initial vector $x^{(0)}$. For $i=1,2, \ldots, \alpha$; $k=1,2, \ldots $ , give also the set $S(i,k)$ and for some $i_{0}$, $S(i_{0},k)=\{1,2,\ldots,n\}$. The sequence of $x^{(k)}$, $k=1,2,\ldots$ , until converges.
Step 1.: *Solution in parallel*. For the *i*th processor, $i=1,2,\ldots,\alpha$: compute $x_{j}^{(i,k)}$, $j\in S(i,k)$ by
  2.6 $$ M_{i}x^{(i,k)}=N_{i}x^{(i,k-1)}+b, $$
  where $x^{(i,k)}=(x_{1}^{(i,k)},\ldots,x_{n}^{(i,k)})^{T}$.
Step 2.: For the $i_{0}$th processor, set
  $$x=\sum_{i=1}^{\alpha}E_{i}^{(k)}x_{i}^{(k)}= \left ( \textstyle\begin{array}{@{}c@{}} \sum_{i=1}^{\alpha}e_{1}^{(i,k)}x_{1}^{(i,k)} \\ \sum_{i=1}^{\alpha}e_{2}^{(i,k)}x_{2}^{(i,k)}\\ \vdots\\ \sum_{i=1}^{\alpha}e_{n}^{(i,k)}x_{n}^{(i,k)} \end{array}\displaystyle \right ). $$
  Solve the following optimization problem:
  2.7 $$\begin{aligned}& \min_{e_{j}^{(i,k)}\in S(k)}\|Ax-b\|_{1} \\& \quad \mbox{s.t. } \sum_{i=1}^{\alpha}E_{i}^{(k)}=I. \end{aligned}$$
  Or
  2.8 $$\begin{aligned}& \|Ax-b\|_{1}\le\bigl\| Ax_{i_{0}}^{(k)}-b\bigr\| _{1} \\& \quad \mbox{s.t. } \sum_{i=1}^{\alpha}E_{i}^{(k)}=I. \end{aligned}$$
Step 3.: Compute
  2.9 $$ x^{(k)}=\sum_{i=1}^{\alpha}E_{i}^{(k)}x_{i}^{(k)}. $$
Step 4.: If $\|Ax^{(k)}-b\|_{1}\leq\epsilon$, stop; otherwise, $k\Leftarrow k+1$, go to Step 1.

### Remark 2.1

The implementation of this method is that at each iteration there are *α* independent problems of the kind (2.6) with $x_{j}^{(i,k)}$, $j\in S(i,k)$ represents the solution to the local problem. The work for each equation in (2.6) is assigned to one processor, and communication is required only to produce the update given in (2.9). In general, some (most) of the diagonal elements in $E_{i}$ are zero and therefore the corresponding components of $x_{j}^{(i,k)}$, $j\in S(i,k)$ need not be calculated.

### Remark 2.2

We may use some optimization methods such as the simplex method (see [19]) to solve an approximated solution satisfying inequality (2.7). Usually, we can compute the optimal weighting at every two or three iterations to replace at each iteration step. Hence, the computational complexity of (2.7) is about $4n^{2}$ or $6n^{2}$ flops.

On the other hand, if $S(i_{0},k)=n$, $S(i,k)=1$, $i\neq i_{0}$, let $x=(1-t)x^{(i_{0},k)}+t\bar{x}^{(k)}$, (2.7) becomes

$$\Vert Ax-b \Vert _{1}= \bigl\Vert Ax^{(i_{0},k)}-b-tA \bigl(x^{(i_{0},k)}-\bar{x}^{(k)}\bigr) \bigr\Vert _{1}= \Vert \bar {b}-\bar{a}t \Vert _{1}. $$

Now, we consider the following programming:

2.10 $$ \min_{x}\sum_{j=1}^{n}|b_{j}-a_{j}x|. $$

### Assumptions

- $b_{j}\geq0$, $j=1,2,\ldots,n$.
- $\frac{b_{1}}{a_{1}}\leq \frac{b_{2}}{a_{2}}\leq\cdots\leq\frac{b_{n}}{a_{n}}$.

### Lemma 2.1

*Let programming* (2.10) *satisfy Assumptions and* $x_{j}=\frac{b_{j}}{a_{j}}$, $j=1,2,\ldots,n$. *Then there exists some* $j_{0}$ *such that* $x_{j_{0}}$ *is the solution of programming* (2.10).

### Proof

As we know, the solution $x^{*}\in[\frac{b_{1}}{a_{1}},\frac{b_{n}}{a_{n}}]$. Let $P=\{\frac{b_{1}}{a_{1}}, \frac{b_{2}}{a_{2}},\ldots,\frac{b_{k}}{a_{k}},\ldots,\frac{b_{n}}{a_{n}}\}$ be a partition of $[\frac{b_{1}}{a_{1}},\frac{b_{n}}{a_{n}}]$, it implies that $\frac{b_{1}}{a_{1}}< \frac{b_{2}}{a_{2}}<\cdots<\frac{b_{k}}{a_{k}}<\cdots<\frac{b_{n}}{a_{n}}$. Thus, we obtain a set of subinterval induced by the partition *P*.

In every subinterval, the function $\sum_{j=1}^{n}|b_{j}-a_{j}x|$ is a linear function, then the minimization point of the linear function is just a partition point. Hence, the lemma has been proved. □

### Corollary 2.2

*Let programming* (2.10) *satisfy Assumptions*. *Then*

$$\min\Biggl\{ \sum_{j=1}^{n} \biggl\vert b_{j}-a_{j}\frac{b_{k}}{a_{k}} \biggr\vert ,\sum _{j=1}^{n} \biggl\vert b_{j}-a_{j} \frac{b_{k+1}}{a_{k+1}} \biggr\vert \Biggr\} \leq\sum_{j=1}^{n}b_{j} $$

*with* $\frac{b_{k}}{a_{k}}\leq 0\leq\frac{b_{k+1}}{a_{k+1}}$.

From Lemma 2.1 we obtain an approximated solution, its complexity is about $4n^{2}$ flops.

## Convergence analysis

In this section, we discuss the convergence of Method 2.1 under the reasonable assumptions.

### Lemma 3.1

*Let* $A=M-N$ *be* *H*-*compatible splitting of the strictly diagonally dominant matrix* *A*, *then*

3.1 $$ \bigl\Vert NM^{-1} \bigr\Vert _{1}< 1. $$

### Proof

From the definition of *H*-compatible splitting, we know that $\langle A\rangle=\langle M\rangle-|N|$.

Let $N=(N_{1}^{T} , N_{2}^{T},\ldots,N_{n}^{T} )^{T}$, $N_{j}=(n_{j1},n_{j2},\ldots ,n_{jn})$. From Property 1.4, it holds that

$$\bigl\Vert NM^{-1} \bigr\Vert _{1}\leq \bigl\Vert |N| \langle M\rangle^{-1} \bigr\Vert _{1}=\max _{1\leq j\leq n} \bigl\vert \bigl(e^{T}|N|\langle M \rangle^{-1}\bigr)_{i} \bigr\vert . $$

Let $e^{T}|N|\langle M\rangle^{-1}=x^{T}$, where $x^{T}=(x_{1},x_{2},\ldots,x_{n})$. We have

$$e^{T}|N|=x^{T}\langle M\rangle. $$

Let $x_{j_{0}}=\max_{1\leq j\leq n}x_{j}$, which implies

$$ \sum_{j=1}^{n}|n_{jj_{0}}|=m_{j_{0}j_{0}}x_{j_{0}}- \sum_{j\neq j_{0}}m_{jj_{0}}x_{j} \geq \biggl(m_{j_{0}j_{0}}-\sum_{j\neq j_{0}}m_{jj_{0}} \biggr)x_{j_{0}},\quad x_{j_{0}}\leq\frac{\sum_{j=1}^{n} |n_{jj_{0}}|}{m_{j_{0}j_{0}}-\sum_{j\neq j_{0}}m_{jj_{0}}}. $$

From the *H*-compatible splitting $\langle A\rangle=\langle M\rangle -|N|$ and the strict diagonal dominance of $\langle A\rangle$, it holds that

$$\frac{\sum_{j=1}^{n} |n_{jj_{0}}|}{m_{j_{0}j_{0}}-\sum_{j\neq j_{0}}m_{jj_{0}}}< 1. $$

Hence, $\|NM^{-1}\|_{1}<1$. □

### Lemma 3.2

*Let* $A=M-N$ *be* *H*-*compatible splitting of the* *H*-*matrix* *A*. *Then there exists a positive diagonal matrix* *D* *such that*

3.2 $$ \bigl\Vert DNM^{-1}D^{-1} \bigr\Vert _{1}< 1. $$

### Proof

There is a positive diagonal matrix *D* such that *DA* is a strictly diagonally dominant matrix since *A* is an *H*-matrix. Thus

3.3 $$ \langle DA\rangle=\langle DM\rangle-|DN|. $$

Let

$$\langle DA\rangle=\bar{A},\qquad \langle DM\rangle=\bar{M},\qquad |DN|=\bar{N}. $$

Then

$$\bar{A}=\bar{M}-\bar{N} $$

is a regular splitting of the strictly diagonally dominant matrix *Ā*. From Lemma 3.1, we know that

$$\bigl\Vert \bar{N}\bar{M}^{-1} \bigr\Vert _{1}< 1. $$

Hence,

$$\bigl\Vert DNM^{-1}D^{-1} \bigr\Vert _{1}= \bigl\Vert \bar{N}\bar{M}^{-1} \bigr\Vert _{1}< 1. $$

 □

### Theorem 3.3

*Let* $A=M_{i}-N_{i}$, $i=1,2,\ldots,\alpha$, *be* *α* *splittings of the* *H*-*matrix* *A*, *and for some* $i_{0}$, *let* $A=M_{i_{0}}-N_{i_{0}}$ *be* *H*-*compatible splitting*. *Assume that* $E_{i}^{(k)}$, $i=1,2,\ldots, \alpha$; $k=1,2,\ldots $ *are yielded by* (2.7) *or* (2.8) *in Method* 2.1. *Then* $\{x^{(k)}\} $ *generated by Method* 2.1 *converges to the unique solution* $x_{*}$ *of* (1.1).

### Proof

Let $\varepsilon^{(k)}=x^{(k)}-x_{*}$. We have

3.4 $$ \varepsilon^{(k+1)}=T_{k}\varepsilon^{(k)}, \qquad \varepsilon_{i}^{(k+1)}=T_{i}\varepsilon^{(k)}, \quad i=1,2,\ldots,\alpha. $$

On the other hand,

3.5 $$ \min_{e^{(i,k)}_{j}\in S(k)} \Vert Ax-b \Vert _{1}=\min _{e^{(i,k)}_{j}\in S(k)} \bigl\Vert A(x-x_{*}) \bigr\Vert _{1}. $$

Let *D* be a positive diagonal matrix such that *DA* is a strictly diagonally dominant matrix. Thus, from (2.8) (or (2.9)) and Lemma 3.2 we know that

$$\begin{aligned} \bigl\Vert DA\varepsilon^{(k+1)} \bigr\Vert _{1} =& \bigl\Vert DAT_{k}\varepsilon ^{(k)} \bigr\Vert _{1} \leq\min_{1\leq i\leq\alpha} \bigl\Vert DAT_{i} \varepsilon^{(k)} \bigr\Vert _{1} \\ \leq& \bigl\Vert DAT_{i_{0}}A^{-1}D^{-1} \bigr\Vert _{1} \bigl\Vert DA\varepsilon^{(k)} \bigr\Vert _{1} = \bigl\Vert DN_{i_{0}}M_{i_{0}}^{-1}D^{-1} \bigr\Vert _{1}\cdot \bigl\Vert DA\varepsilon^{(k)} \bigr\Vert _{1} \\ \le& \bigl\Vert D|N_{i_{0}}|\langle M_{i_{0}} \rangle^{-1}D^{-1} \bigr\Vert _{1}\cdot \bigl\Vert DA\varepsilon^{(k)} \bigr\Vert _{1} \leq r \bigl\Vert DA\varepsilon^{(k)} \bigr\Vert _{1} \\ \leq&\cdots\leq r^{k+1} \bigl\Vert DA\varepsilon^{(0)} \bigr\Vert _{1}, \end{aligned}$$

where $r=\|D|N_{i_{0}}|\langle M_{i_{0}}\rangle^{-1}D^{-1}\|_{1}<1$.

Thus, $\lim_{k\rightarrow\infty}\|DA\varepsilon^{(k+1)}\|_{1} =0$, which implies that

$$\lim_{k\rightarrow\infty}\varepsilon^{(k+1)}=0. $$

We have completed the proof of the theorem. □

## Numerical experiments

In this section, a test problem to assess the feasibility and effectiveness of Method 2.1 in terms of both iteration number (denoted by IT) and computing time (in seconds, denoted by CPU) is given. All our tests are started from zero vector and terminated when the current iterate satisfied $\|r^{(k)}\|_{1}<10^{-6}$, where $r^{(k)}$ is the residual of the current, say *k*th iteration or the number of iterations is up to 20,000. For the latter the iteration is failing. We solve (2.7) or (2.8) in the optimization step by the simplex method (see [19]).

### Problem

Consider the generalized convection-diffusion equations in a two-dimensional case. The equation is

4.1 $$ -\frac{\partial^{2} u}{\partial x^{2}}-\frac{\partial^{2} u}{\partial y^{2}}+q\cdot\exp(x+y)\cdot x\cdot \frac{\partial u}{\partial x}+q\cdot\exp(x+y)\cdot y\cdot\frac{du}{dy}=f $$

with the homogeneous Dirichlet boundary condition. We use the standard Ritz-Galerkin finite element method by $P_{1}$ conforming triangular element to approximate the following continuous solutions $u=x\cdot y\cdot(1-x)\cdot(1-y)$ in the domain $\Omega=[0,1]\times[0,1]$, the step-sizes along both *x* and *y* directions are the same, that is, $h=\frac{1}{2^{m}}$, $m=5,6,7$. Let $q=1$.

After discretization, the matrix *A* of this equation is given by

$${A}=\left [ \textstyle\begin{array}{@{}c@{\quad}c@{\quad}c@{\quad}c@{\quad}c@{}} A_{11}&B_{12}& & & \\ C_{21}&A_{22}&B_{23}& &\\ &\ddots&\ddots&\ddots&\\ & & C_{p-1,p-2}& A_{p-1,p-1}&B_{p-1,p}\\ & & &C_{p,p-1}&A_{p,p} \end{array}\displaystyle \right ]\in\mathbf{R}^{n\times n}, $$

where $A_{i,i}$, $i=1,\ldots, p$, are *s*-by-*s* nonsymmetric matrices and $B^{T}_{i,i+1}\neq C_{i+1,i}$. Thus, $n=s\cdot p$ and $s=p=2^{m}=32,64,128$ from the above.

Let

$${A}={D}-{L}-{U}, $$

where *D* is a block diagonal matrix, *L* is the strictly block lower triangle matrix, *U* is the strictly block upper triangle matrix.

We construct the multisplitting as follows:

- The block Jacobi splitting (denoted by BJ)
  $${A}={M}_{1}-{N}_{1}, \qquad {M}_{1}={D}. $$
- The block Gauss-Seidel splitting I (denoted by BGS-I)
  $${A}={M}_{2}-{N}_{2},\qquad {M}_{2}= {D}-{L}. $$
- The block Gauss-Seidel splitting II (denoted by BGS-II)
  $${A}={M}_{2}-{N}_{2},\qquad {M}_{2}= {D}-{U}. $$

The three weighting matrices are chosen as follows:

$$\begin{aligned}& E_{1}=\operatorname{diag}(\omega I, \omega I), \\& E_{2}=\operatorname{diag}(\beta I, 0), \\& E_{3}=\operatorname{diag}(0,\gamma I), \end{aligned}$$

where *I* is the $\frac{n}{2} \times\frac{n}{2}$ identity matrix.

In the first results presented in Table 1 we show the spectral radius of the corresponding iteration matrix for the classical iterative methods (a)-(c). It is well known that an iterative method is convergent when the spectral radius of the corresponding iteration matrix is less than one. We can see that the numbers listed in Table 1 approximate to 1 such that these iterative methods converge to the unique solution of (4.1) slowly. However, the behavior of the multisplitting method based on three splittings (a)-(c) can be improved by self-adaptive weightings, and the results are shown in Tables 2 and 3.

**Table 1:** **Radii of the classical iterative methods**

***n***	**BJ**	**BGS-I**	**BGS-II**
32 × 32	0.9923	0.984	0.9847
64 × 64	0.9980	0.9960	0.9961
128 × 128	0.9995	0.9990	0.9991

**Table 2:** **The comparison of computational results among the BGS-I, BGS-II, Method** **2.1**

***n***		**BGS-I**	**BGS-II**	**Method** **2.1**
32 × 32	IT	866	869	653
	CPU(s)	3.465	3.838	2.5130
64 × 64	IT	3,353	3,358	2,314
	CPU(s)	64.493	78.830	23.2740
128 × 128	IT	13,191	13,202	6,799
	CPU(s)	1,203.512	1,367.604	365.7850

Note: $k_{\mathrm{opt}}=2$ can be chosen in Method 2.1.

**Table 3:** **The comparison of computational results between Method** **2.1** **and Basic Method**

***n***		**Method ** **2.1**	**B-Meth 1**	**B-Meth 2**	**B-Meth 3**
32 × 32	IT	653	1,308	1,226	1,175
	CPU(s)	2.5130	4.4435	3.3955	3.1638
64 × 64	IT	2,314	4,571	4,601	4,489
	CPU(s)	23.2740	57.1624	57.1404	55.3278
128 × 128	IT	6,799	17,865	17,662	17,877
	CPU(s)	365.7850	1,048.6420	1,047.1182	1,040.2860

Here, we denote the basic parallel multisplitting iterative method with fixed weighting matrix by B-Meth (see [1]). In Basic Methods, we propose three groups of weighting matrices, which are generated by random selection. Thus, the corresponding parallel multisplitting iterative methods are denoted by B-Meth 1, B-Meth 2, B-Meth 3, respectively.

The speed-up is defined in the following:

$$\mbox{speed-up}=\frac{\mbox{CPU of the Basic Methods}}{ \mbox{CPU of Method 2.1}}. $$

From the above numerical experiments, it is obtained that the average speed-up of the new parallel multisplitting iterative method (Method 2.1) is about 2.3 (the average value of all computational results by Basic Methods).

## Conclusion

The parallel multisplitting iterative method with the self-adaptive weighting matrices has been proposed for the linear system of equations (1.1) when the coefficient matrix is an *H*-matrix. The convergence theory is established for the parallel multisplitting method with self-adaptive weightings. The numerical results show that the new parallel multisplitting iterative method with the self-adaptive weightings is effective.
